# Evaluating the impact of view position in X-ray imaging for the classification of lung diseases

**DOI:** 10.1007/s13246-025-01579-1

**Published:** 2025-07-28

**Authors:** Aya Hage Chehade, Nassib Abdallah, Jean-Marie Marion, Mohamad Oueidat, Pierre Chauvet

**Affiliations:** 1 LARIS, University of Angers, Angers, France; 2 LaTIM, INSERM, UMR 1101, University of Brest, Brest, France; 3 University Hospital of Brest, Brest, France; 4 Faculty of Technology, Lebanese University, Beirut, Lebanon

**Keywords:** ChestX-ray14 dataset, Convolutional neural network, Hierarchical cluster analysis, Image classification, Image processing, Spatial attention mechanism, Unsharp masking

## Abstract

Clinical information associated with chest X-ray images, such as view position, patient age and gender, plays a crucial role in image interpretation, as it influences the visibility of anatomical structures and pathologies. However, most classification models using the ChestX-ray14 dataset relied solely on image data, disregarding the impact of these clinical variables. This study aims to investigate which clinical variable affects image characteristics and assess its impact on classification performance. To explore the relationships between clinical variables and image characteristics, unsupervised clustering was applied to group images based on their similarities. Afterwards, a statistical analysis was then conducted on each cluster to examine their clinical composition, by analyzing the distribution of age, gender, and view position. An attention-based CNN model was developed separately for each value of the clinical variable with the greatest influence on image characteristics to assess its impact on lung disease classification. The analysis identified view position as the most influential variable affecting image characteristics. Accounting for this, the proposed approach achieved a weighted area under the curve (AUC) of 0.8176 for pneumonia classification, surpassing the base model (without considering view position) by 1.65% and outperforming previous studies by 6.76%. Furthermore, it demonstrated improved performance across all 14 diseases in the ChestX-ray14 dataset. The findings highlight the importance of considering view position when developing classification models for chest X-ray analysis. Accounting for this characteristic allows for more precise disease identification, demonstrating potential for broader clinical application in lung disease evaluation.

## Introduction

Lung diseases pose a significant risk to global health, as they are the primary cause of health-related fatalities across the globe. Lung disease is categorized as the third leading cause of mortality globally [1], with an estimated total of five million deaths per year [2]. According to the World Health Organization, pneumonia is the single largest infectious cause of death in children worldwide, with a reported 740,180 children under five years old dying from the disease in 2019 [3]. Consequently, early diagnosis of lung diseases is crucial for effective treatment and reduction of mortality.

Various types of imaging such as X-ray and CT scans are performed to diagnose lung diseases. Chest radiography, or chest X-ray (CXR), is the most common medical imaging technique due to its accessibility, simplicity and cost-effectiveness [4, 5], playing a vital role in diagnosing various lung diseases, including pneumonia [6].

However, manual observation of CXR is time-consuming and challenging for radiologists due to the complex nature of chest X-rays [7] and the presence of various lung diseases, some of which may have similar visual characteristics.

Recently, artificial intelligence (AI) technologies have been known for their ability to examine data faster, improve the efficiency of CXR analysis for the detection and classification of lung diseases and make decisions. Machine learning (ML) and deep learning (DL) can play a significant role in accurate clinical diagnosis [8–10]. More recently, DL has been widely investigated due to its general applicability to problems involving automated feature extraction and classification. Specifically, Convolutional Neural Network (CNN) are widely used in image classification and object detection [23–25]. CNN models automatically extract hierarchical features from simple to complex by convoluting the original input images with high-dimensional filters that automatically gather information of the structure embedded in the image [26]. The advent of Deep Learning and the release of the large-scale CXR dataset [11] have spurred numerous studies on developing deep learning-based techniques for disease detection and classification.

Moreover, metadata associated with radiographic images, such as the view position of the X-ray (i.e. whether the X-ray images were acquired in the posterior-anterior (PA) or anterior-posterior (AP) position), the age and gender of the patient, are crucial for the accurate interpretation of CXR images by physicians [12] as well as for the decision-making process [13].

Indeed, this additional information provides valuable context that can significantly affect not only the interpretation of the images but also the decision-making regarding the presence of a pathology. For instance, the patient’s age influences the evaluation criteria due to anatomical and physiological changes in children compared to adults, as certain pathologies are more prevalent or manifest differently across age groups. Similarly, the patient’s gender impacts the evaluation of images, considering the anatomical and physiological differences between men and women, which can affect both the appearance and interpretation of radiological signs of diseases. The view position of an image can reveal specific anatomical details that are crucial to correctly identify abnormalities or pathologies [14]. Indeed, PA views provide a clearer and more accurate image of the lungs and thorax, reducing the heart’s enlargement and enabling a more precise evaluation of lung tissues. In contrast, AP views can distort the perception of pulmonary abnormalities, making it more challenging to detect subtle details or accurately assess the size and shape of lung lesions.

Previous studies using the ChestX-ray14 dataset have focused solely on simple image analysis, overlooking the clinical information available within the dataset. For instance, Yao et al. [10] proposed an approach based on Long Short-Term Memory (LSTM) networks that treats the classification problem as a sequence prediction task. Their model is based on the DenseNet architecture and incorporates four ConvBlocks within each of the four DenseBlocks to keep the total number of parameters small, along with three TransitionBlocks. The model was trained from scratch to capture application-specific features. Rajpurkar et al. [15] proposed a 121-layer deep transfer learning model called CheXNet for pneumonia classification from CXR images. In addition to classifying pneumonia, they also demonstrated that their model can determine the most symptomatic areas of pneumonia in a CXR. They compared their performance with that of radiologists and demonstrated that the model can outperform the average radiologist’s performance in diagnosing pneumonia. Souid et al. [16] proposed the classification and prediction of lung pathologies in CXRs using the MobileNet v2 model [17], supplemented by CNN layers. To detect diseases from the ChestX-ray14 dataset, Mann et al. [19] used three convolutional neural networks, namely DenseNet-121, ResNet-50, and EfficientNet-B1. Experimental results showed that DenseNet-121 outperformed the other two models. DenseNet-121 was used as a feature extractor and a more intricate fully connected layer was trained on it. Only fully connected layers were modified during the training process and the rest of the DenseNet-121 architecture remained unchanged.

In a unique study, Baltruschat et al. demonstrated that incorporating non-image data into model features led to an improvement in performance [18].

The objective of this study is to explore which clinical variables impact the visualization of lung diseases in CXR images and, consequently, influence the classification performance using the ChestX-ray14 dataset. Specifically, this study highlights the importance of the view position variable (AP/PA) by evaluating its impact on the classification of lung diseases.

The study proposed an approach that leverages the clinical data available in the ChestX-ray14 dataset by examining both images and their associated clinical variables to identify links between them. This analysis identified groups that exhibit similar view position characteristics, specifically the AP and PA views. Furthermore, the study further demonstrated the importance of the view position variable by assessing its impact on lung disease classification. A spatial attention-based CNN model was developed for each group, allowing the model to weight features based on their importance, and the impact of the attention mechanism on lung disease classification was evaluated. Finally, experiments conducted on the ChestX-ray14 dataset demonstrated that the proposed approach outperforms state-of-the-art methods.

## Material and methods

### Dataset

The ChestX-ray14 dataset [11] is extracted from the clinical PACS databases in the hospitals affiliated to National Institutes of Health Clinical Center and includes 112,120 chest X-ray images from 30,805 unique patients. Each CXR is labeled with binary labels for 14 different diseases. The CXR is labeled as “no finding” if none of the diseases present in the ChestX-ray14 dataset were detected. All CXRs are in PNG format and have a size of $1024 \times 1024$. The dataset also contains other data such as the gender and age of the patient and the view position of the X-ray (i.e. whether the X-ray images were acquired in the posterior-anterior (PA) or anterior-posterior (AP) position). Table 1 provides a summary of the data available in the ChestX-ray14 dataset, including the number of images with and without diseases, along with other associated clinical variables.

**Table 1:** Summary of the data available in the ChestX-ray14 dataset

Total number of images	112,120
Number of unique patients	30,805
Number of diseases	14
Image size	$1024 \times 1024$
Number of images with diseases	51,759
Number of no finding images	60,361
View positions	Posterior-Anterior (PA) or Anterior-Posterior (AP)
Other clinical variables	Gender, age

In this dataset, 322 images are labeled as pneumonia and 60,361 images are no finding. To address class imbalance, the no finding images were down-sampled to obtain 354 samples. A slightly higher number of no finding images was retained compared to disease images to better reflect the general distribution of the dataset, where no finding cases are typically more prevalent. Therefore, experiments were conducted on a total of 676 images along with their corresponding clinical variables.

The pipeline was also tested on two additional diseases, namely consolidation and infiltration. In the ChestX-ray14 dataset, 1310 images are labeled as consolidation, and 9547 images are labeled as infiltration. To perform the binary classification of these two pathological conditions and to address the class imbalance problem, the no finding images were down-sampled to 1441 for the classification of consolidation and 10,502 for the classification of infiltration. As a result, the binary classification of consolidation and no finding was conducted on a total of 2751 images, and the binary classification of infiltration and no finding was performed on a total of 20,049 images.

These three diseases were selected due to their lower classification performance in previous studies using the ChestX-ray14 dataset. The proposed approach was subsequently evaluated across all 14 diseases in the ChestX-ray14 dataset using the best-performing model.

**Fig. 1:**
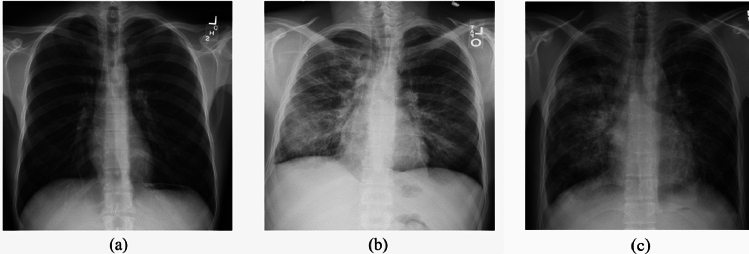
Sample images from the ChestX-ray14 dataset: **a** Normal CXR, **b** Pneumonia case, and **c** Infiltration case. The pathologies in (**b**, **c**) can be distinguished from the normal case in (**a**) by the presence of abnormal opacities in the pulmonary lobes. However, **b**, **c** are not easily distinguishable from each other due to similar radiographic appearances

Figure 1 illustrates samples of CXRs from the ChestX-ray14 dataset, including a normal case, a pneumonia case, and an infiltration case. The pathological cases are characterized by the presence of abnormal opacities in the lungs. However, it remains challenging to characterize the specific pathology based solely on these visual features, as different lung conditions can present with similar radiographic appearances.

### Overview of the methodology

**Fig. 2:**
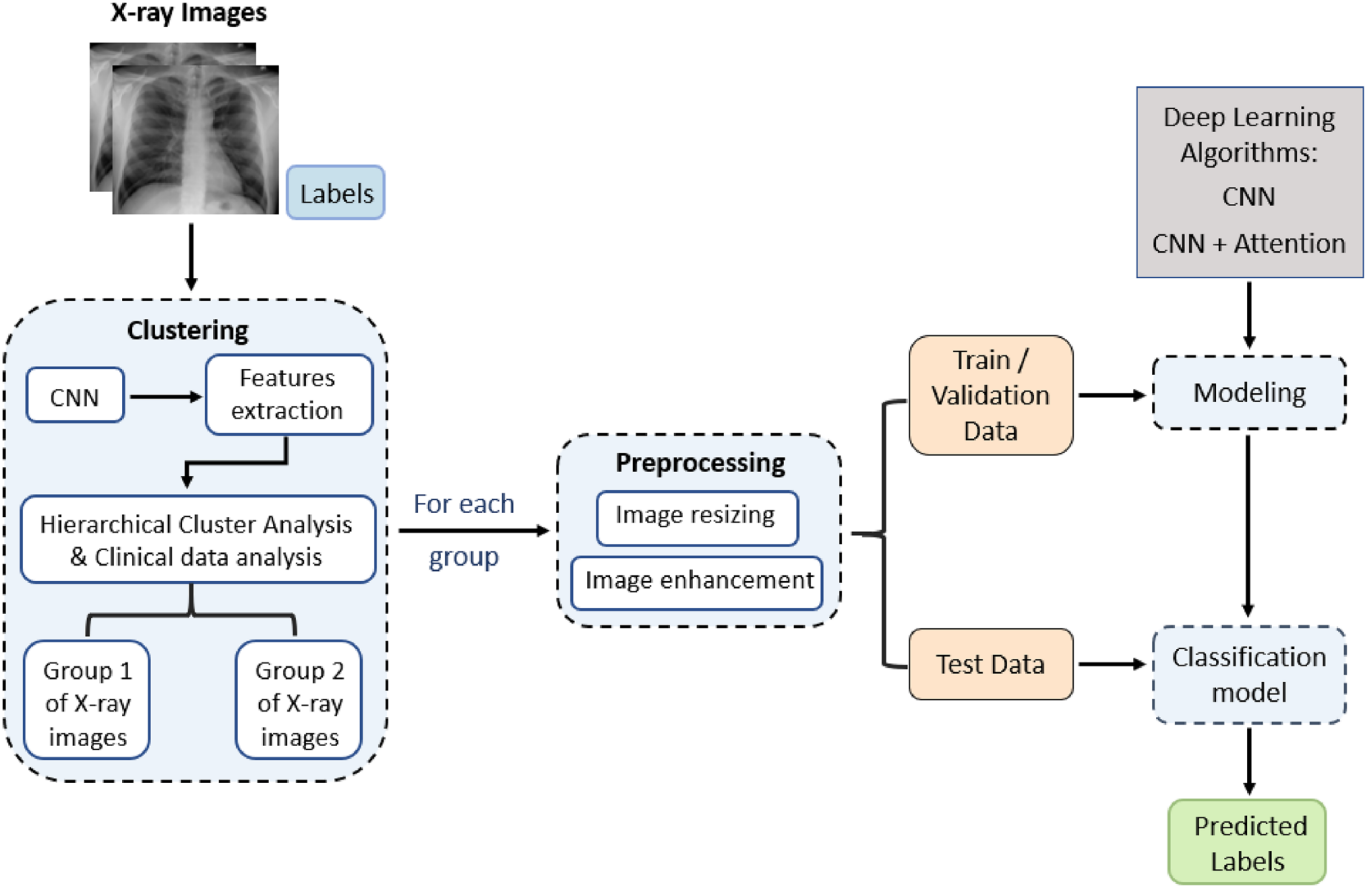
Overview of the proposed methodology for X-ray images classification

Figure 2 shows an overview of the methodology used for classifying the pathological conditions and no finding cases on the basis of X-ray images. Chest X-ray images with their corresponding clinical variables were used. An unsupervised clustering method which is the Hierarchical Cluster Analysis (HCA) was used to to identify links between the inherent nature of the images and the available clinical variables. The resulting clusters were analyzed and grouped on the basis of their common clinical variables: one group with a majority of PA views and another group with a majority of AP views. The main advantage of using HCA is that it allows a better view of the subgroups within the data and a better understanding of the clinical variables within the population under study. Then, for each group, images were resized to $224 \times 224$ pixels, and normalized in an interval [0; 1]. The Unsharp Masking method was used to enhance the contrast of images. After that, 20% of the data in each group was used as test data, 10% as validation data and 80% was devoted to training the data (randomly chosen). The data was stratified to ensure proportional representation of both classes across the training, validation, and test sets. Thereafter, classification models based on CNNs and spatial attention mechanism were tested for each group. The test set images were used to evaluate model performance, which was quantified by the Area Under the Curve (AUC). The AUC was chosen as the primary evaluation metric to compare the results with previous studies using the same dataset. In the literature, AUC is the standard evaluation metric for evaluating lung disease classification models in the chestXRay challenge, which justifies its choice in this study. Additionally, to further validate the results, additional evaluation metrics such as Accuracy, Precision, Recall, and F1-score were calculated for the classification of the three pathological conditions using the model that produced the best results. The programming language used is Python with the implementation of the following libraries: numpy, pandas, matplotlib, scipy, tensorflow, scikit-learn and scikit-image.

### Hierarchical cluster analysis (HCA)

The proposed approach begins with unsupervised clustering using HCA to identify clusters of images that share similar characteristics, without presupposing the number of clusters [20]. By identifying homogeneous image groups, it provides a clearer understanding of the subgroups present in the data and enables a comprehensive analysis by exploring the relationship between the inherent nature of the images and the clinical variables available in the ChestX-ray14 dataset, thus avoiding a narrow focus on image data analysis alone.

**Fig. 3:**
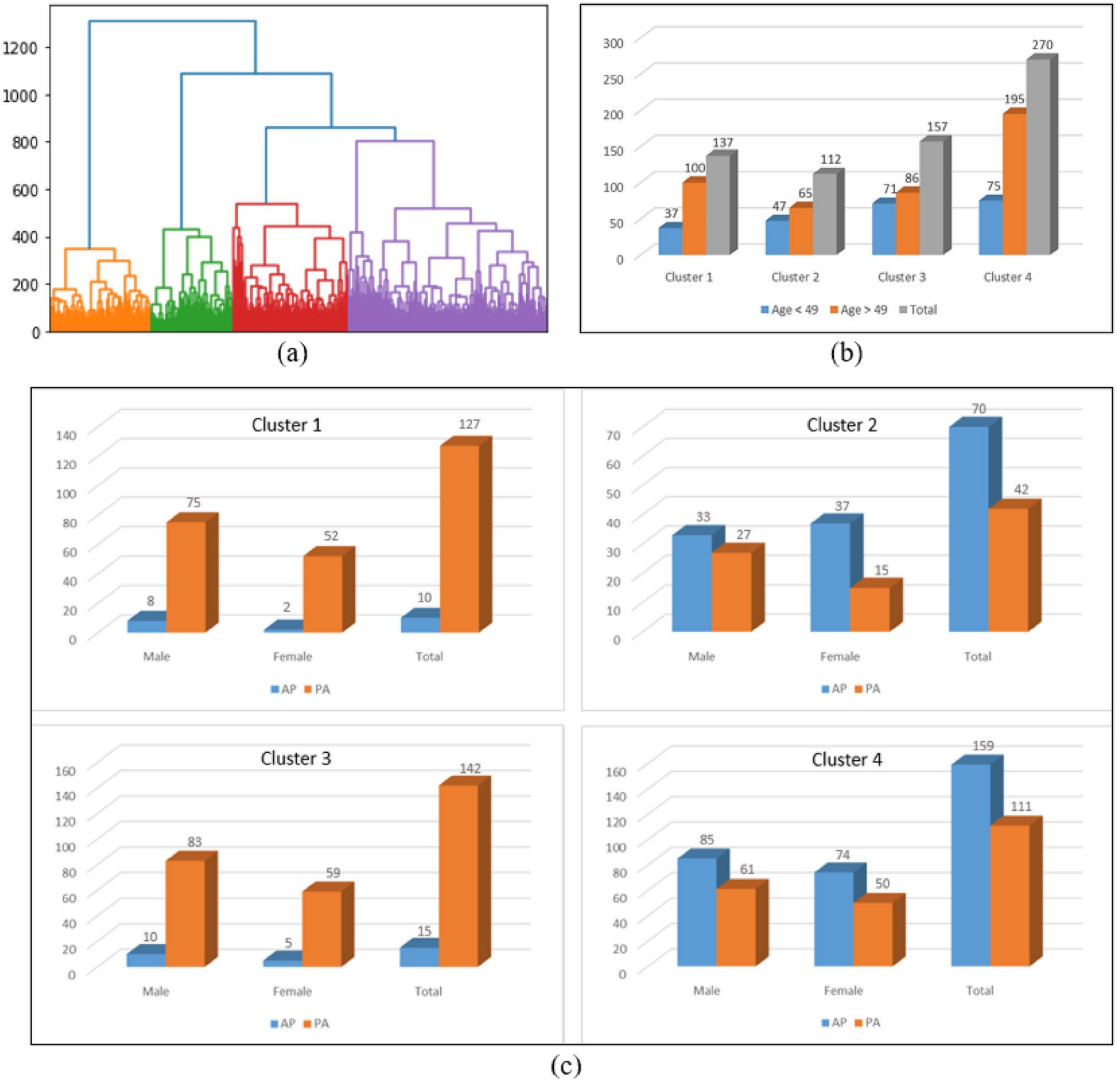
**a** Dendrogram generated by the HCA for the images of pneumonia and no finding, **b** Distribution of images based on patients’ age in each cluster (Y-axis represents the number of images; blue bars correspond to images from patients aged 49 years, and orange bars to images from patients aged 49 years). **c** Distribution of images based on gender and view position (AP/PA) within each cluster (Y-axis represents the number of images; blue bars indicate AP view position, and orange bars indicate PA view position)

First, a CNN with five convolutional layers to extract features from the images. These features are then used as input data for the HCA to form clusters. The result of this analysis is typically represented in a dendrogram, where similar samples are grouped together based on their characteristics. The dendrogram generated by the HCA classification, as shown in Fig. 3a, suggests a four-group data partition for the images of pneumonia and no finding.

The clinical variables of the patients corresponding to the images in each cluster were analyzed. From Fig. 3b, c, it is observed that in all clusters, there are more images for patients over 49 years old (where 49 corresponds to the median age of patients in the ChestX-ray14 dataset) and more images for male patients than female patients. However, the image view position shows more variability among clusters, indicating different technical preferences that influence the composition of the cluster. Consequently, clusters with similar dominant image view position characteristics were grouped, forming two new groups: one group with a majority of PA views (91.5%) and another group with a majority of AP views (60%). This distinction is clinically significant, as the view position can affect the visibility of anatomical structures and the detection of abnormalities, which is crucial for accurate diagnosis.

In the subsequent analysis, the impact of view position on lung disease classification was evaluated. For this purpose, preprocessing and classification models were applied to each of the two newly formed groups, and the weighted AUC was calculated.

### Image preprocessing

Following clustering, Unsharp Masking (UM) method is used to enhance the contrast, and thus sharpen the original image, allowing the emphasis of textures and details [22]. Indeed, image preprocessing is crucial to improve image quality, highlight important details in low contrast areas and extract relevant information from CXR images to make them more suitable for the learning algorithm [21].

The main concept of the UM method is to subtract the original image by a blurred version of the image itself, thus retaining only the blurred edges. The effectiveness of the Unsharp Masking technique is influenced by the choice of radius and amount parameters. These parameters play a crucial role in determining the extent of sharpening applied to the image.

For the blurring step in the Unsharp Masking technique, various image filter methods can be used, although the Gaussian filter is commonly used. The radius parameter in the unsharp masking filter refers to the sigma parameter of the Gaussian filter. The radius parameter controls the degree of blurring of the original image, determining the number of pixels on either side of an edge that will be affected by sharpening. The amount parameter is a scaling factor that controls the intensity of the sharpening effect applied to the edges. Therefore, by adjusting these two parameters appropriately, it is possible to achieve the desired outcome in terms of sharpening and emphasizing the details in the image.

The typical formula used for unsharp masking is as follows:

1 $$\begin{aligned} I_{\text {sharp}} = I + \alpha \left( I - G_{\sigma }(I) \right) \end{aligned}$$

where $I_{\text {sharp}}$ is the enhanced image, $I$ is the original image, $\alpha$ is an enhancement factor that controls the amount of sharpening applied, and $G_{\sigma }(I)$ is a blurred version of the image obtained using a Gaussian filter with the sigma ($\sigma$) parameter.

**Fig. 4:**
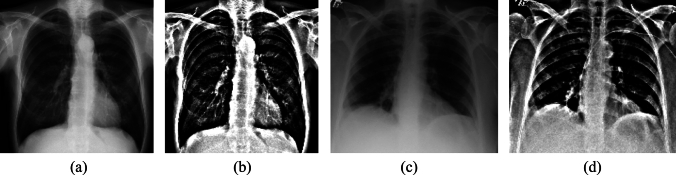
A sample of pneumonia X-ray image: **a** original image from the first group, **c** original image from the second group, **b**, **d** sharpened images using Unsharp Masking with the best parameters for each group

In this study, in order to choose the best parameters for the unsharp masking method for each group, a sensitivity study was conducted on the parameters, testing various radius and amount values. The results showed that the optimal values of the parameters 4 (radius) and 6 (amount) for the first group, and 5 (radius) and 8 (amount) for the second group, as the model demonstrated the best performance for pneumonia classification in each group using these values. Therefore, these values are used in the rest of the study. Figure 4 illustrates the result of enhancing an X-ray image using the Unsharp Masking method under the specified conditions.

### Classification

In response to the challenge of lung diseases appearing in small disease-specific areas, a CNN-Attention model was developed, combining the strengths of both CNN and spatial attention mechanism. While CNNs effectively capture local features and patterns in images, the attention mechanism enables the model to focus on relevant regions of the image called areas of interest, rather than considering the input as a whole.

**Fig. 5:**
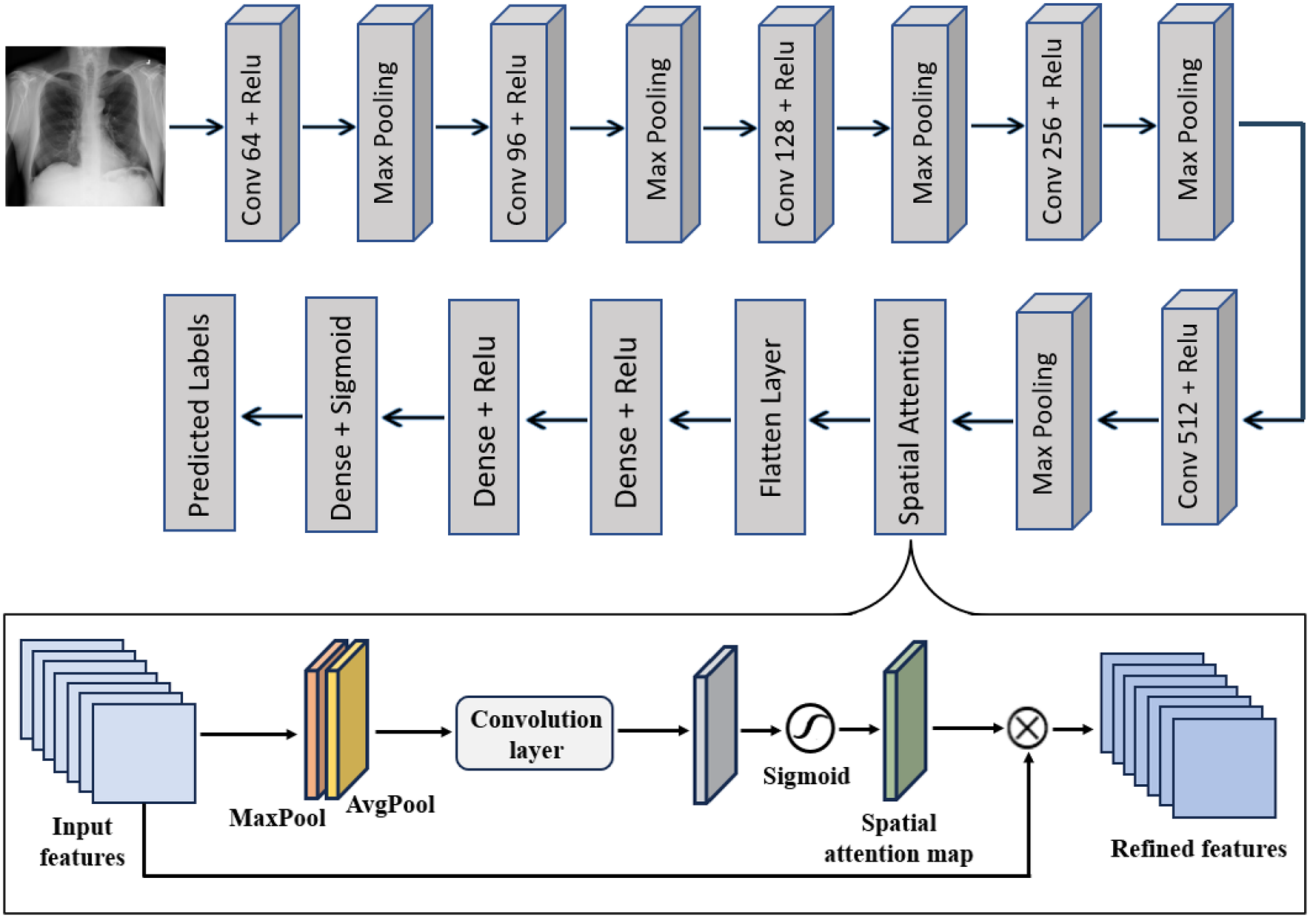
Architecture of the proposed CNN-Attention model

The CNN-Attention model (presented in Fig. 5) applies the spatial attention mechanism to the last convolutional layer’s feature map. In this study, a sequential CNN model was designed, consisting of five Conv2D layers, each having 64, 96, 128, 256 and 512 filters respectively with $3 \times 3$ filter size. Each of these layers is followed by a ReLU activation function. Additionally, there are five Max Pooling layers which are employed to reduce the size of the feature maps to half using a $2 \times 2$ filter. Then, a flatten layer followed by two dense layers with 500 hidden neurons, and two dropout layers are used. The dropout rate is set as 0.5 for the last two fully connected layers. Finally, a dense output layer with two neurons and a sigmoid activation function that classifies the output image to pneumonia or normal.

Additionally, the spatial attention begins by applying average and maximum pooling operations on feature maps, producing two distinct 2D feature maps. These global features are then concatenated to create a combined representation that captures both mean and maximum value information. The combined representation of global features is used as input for a 2D convolution layer with a kernel size of 7. This convolution layer produces an attention map as output. The sigmoid activation function is applied to the attention map to compress its values between 0 and 1. This yields an attention map that represents the attention weights for each image region. These attention weights are element-wise multiplied to the extracted features of the last convolutional layer, thus highlighting the important parts and attenuating the less relevant ones. The weighted features would then be used for further model processing.

**Fig. 6:**
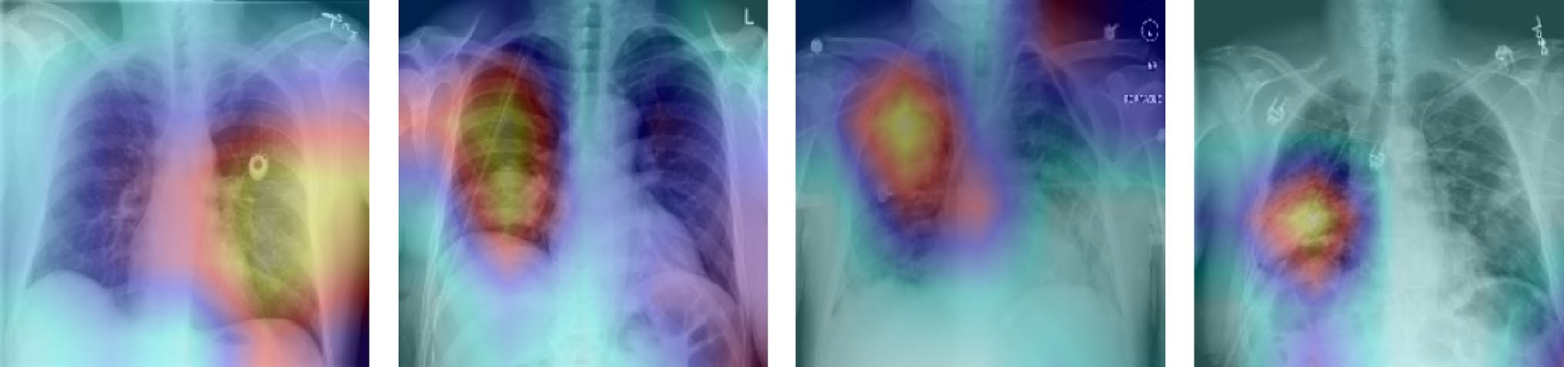
Visualization examples of the disease localization on the test images of pneumonia, consolidation and infiltration. Higher response is indicated with yellow, and lower response with blue

Therefore, the attention mechanism analyzes the feature maps produced by the CNN and assigns weights to different spatial locations based on their importance, allowing the model to focus on the most important and informative image regions while ignoring non-relevant areas. Figure 6 illustrates an example of the resulting attention map, highlighting the regions of the image that are most relevant for disease localization.

The classification model was trained specifically on each of the two groups: the group with a majority of PA views and the group with a majority of AP views. The network was trained with mini-batches of size 16. The ADAM [27] optimizer was used as an optimization algorithm during training with a learning rate of $10^{-4}$. To regularize the network, early stopping strategy was performed with a patience of 5 epochs, which is a method used to detect convergence of training and thus prevent the networks from overfitting. To compile the model, ‘binary_crossentropy’ was used as a loss function.

The model’s performance was assessed by calculating the AUC [28] for each group, which indicates how well the model is able to distinguish between classes. To obtain an overall AUC for the entire model across all groups, a weighted AUC was computed, accounting for the different distributions of the two groups. The weighted AUC is calculated by multiplying each group’s AUC by its respective proportion (relative to the total number of samples) and summing these values. This weighted AUC gives a more accurate overall performance measure by accounting for the varying sample sizes in the different groups. In addition, the accuracy, precision, recall, and F1 score were calculated for the classification of the three pathological conditions using the best performing model.

## Results

The models were evaluated on the test data to determine their performance. To ensure the stability of the results, ten different splits of the data into training, validation, and testing sets were performed, and the results were presented in terms of mean and standard deviation.

Across all results, the highest AUC was obtained when applying UM with the CNN-Attention model for each of the two groups with the same training and test dataset. This model achieved a weighted AUC of 0.8176 for the classification of pneumonia. Table 2 presents the results in detail.

Additionally, when the proposed approach was applied to two other diseases, namely consolidation and infiltration, the CNN-Attention model, incorporating the clustering step and UM as preprocessing, achieved the highest weighted AUC for each disease, with values of 0.8287 and 0.7724, respectively.

Table 3 presents the results of the binary classification of the three diseases by applying the UM with the CNN-Attention model, with and without the clustering step.

Table 4 presents additional evaluation metrics for the best-performing model, which is the CNN-Attention model with the application of the clustering step and the UM method, for the binary classification of the three lung diseases.

As shown in Table 5, the proposed approach outperforms existing methods in the literature for the binary classification of all the lung diseases within the ChestX-ray14 dataset.

**Table 2:** AUC of each group and the weighted AUC with and without UM for the binary classification of pneumonia

AUC	Without UM		With UM	
	CNN	CNN + Attention	CNN	CNN + Attention
Group 1	0.7814 $\pm\, {0.014}$	0.7985 $\pm\, {0.015}$	0.8067 $\pm\, {0.014}$	0.8195 $\pm\, {0.014}$
Group 2	0.7756 $\pm\, {0.015}$	0.7943 $\pm\, {0.016}$	0.7939 $\pm\, {0.012}$	0.8152 $\pm\, {0.015}$
Weighted AUC	0.7789 $\pm\, {0.014}$	0.7967 $\pm\, {0.015}$	0.8011 $\pm\, {0.013}$	**0**.**8176** $\pm\, {0.014}$

Significant value is in bold

The results are presented in terms of mean and standard deviation

**Table 3:** AUC of the binary classification of the three diseases with and without the clustering step

Disease	Unsharp Masking + CNN-Attention	
	With clustering	Without clustering
Pneumonia	0.8176 $\pm\, {0.014}$	0.7941 $\pm\, {0.015}$
Consolidation	0.8287 $\pm\, {0.013}$	0.8035 $\pm\, {0.015}$
Infiltration	0.7724 $\pm\, {0.015}$	0.7568 $\pm\, {0.014}$

The values for “With Clustering” represent weighted AUC, while the values for “Without Clustering” represent standard AUC. The results are presented in terms of mean and standard deviation

**Table 4:** Detailed results of the CNN-Attention model with the application of the clustering step and the unsharp masking method for the binary classification of the three lung diseases

Binary classification	Accuracy	Precision	Recall	F1-score
Pneumonia	0.7922 $\pm\, {0.015}$	0.7884 $\pm\, {0.016}$	0.7877 $\pm\, {0.014}$	0.7881 $\pm\, {0.015}$
Consolidation	0.8063 $\pm\, {0.014}$	0.8021 $\pm\, {0.015}$	0.8013 $\pm\, {0.014}$	0.8017 $\pm\, {0.015}$
Infiltration	0.7689 $\pm\, {0.016}$	0.7672 $\pm\, {0.015}$	0.7656 $\pm\, {0.016}$	0.7664 $\pm\, {0.016}$

The results are presented in terms of mean and standard deviation

**Table 5:** Comparison of AUC results between the best approach and other existing methods in the literature using the ChestX-ray14 dataset

Disease	Yao et al. [10]	Baltruschat et al. [18]	Souid et al. [16]	Yang et al. [29]	The proposed approach	Improvement vs best literature value (%)
Atelectasis	0.772	0.767	*0.8*	0.788	**0.8365** $\pm\, {0.012}$	+3.65
Cardiomegaly	*0.904*	0.883	0.88	0.875	**0.9148** $\pm\, {0.014}$	+1.08
Consolidation	*0.788*	0.749	0.78	0.756	**0.8287** $\pm\, {0.013}$	+4.07
Edema	*0.882*	0.846	0.88	0.854	**0.9021** $\pm\, {0.016}$	+2.01
Effusion	0.859	0.822	*0.87*	0.837	**0.8839** $\pm\, {0.013}$	+1.39
Emphysema	0.829	0.895	0.89	*0.934*	**0.9443** $\pm\, {0.014}$	+1.03
Fibrosis	0.767	0.816	0.78	*0.849*	**0.8657** $\pm\, {0.012}$	+1.67
Hernia	0.914	0.937	0.78	*0.944*	**0.9512** $\pm\, {0.016}$	+0.72
Infiltration	0.695	0.694	0.7	*0.722*	**0.7724** $\pm\, {0.015}$	+5.04
Mass	0.792	0.82	0.83	*0.845*	**0.8582** $\pm\, {0.013}$	+1.32
Nodule	0.717	0.747	0.73	*0.796*	**0.8135** $\pm\, {0.014}$	+1.75
Pleural_Thickening	0.765	0.763	0.77	*0.789*	**0.8271** $\pm\, {0.015}$	+3.81
Pneumonia	0.7113	0.714	*0.75*	0.734	**0.8176** $\pm\, {0.014}$	+6.76
Pneumothorax	0.841	0.84	*0.87*	0.862	**0.8892** $\pm\, {0.013}$	+1.92

Significant values are in bold. The italic values represent the best results reported in the literature

## Discussion

To evaluate the impact of the preprocessing step, model performance was compared with and without the application of UM. As shown in Table 2, applying UM consistently improved the weighted AUC across all models. For example, the AUC of the CNN model increased from 0.7789 to 0.8011, representing an improvement of 2.22%, while that of the Attention-based CNN model increased from 0.7967 to 0.8176, corresponding to an increase of 2.09%, demonstrating the effectiveness of this preprocessing step. Indeed, UM enhances image contrast and sharpness, emphasizing texture and details that may be critical for classification. Therefore, UM is used as a preprocessing step for the rest of the study. Furthermore, as clearly illustrated from the results in Table 2, the classification results with the use of UM and Attention-based CNN model are better than with the CNN model. Specifically, the weighted AUC increased from 0.8011 with the CNN model to 0.8176 with the Attention-based CNN model, resulting in a 1.65% improvement. This confirms the benefit of both the preprocessing step and the attention mechanism, leading to enhanced classification performance, particularly for the binary classification of pneumonia and no finding images.

Overall, the CNN-Attention model holds promise for addressing the challenge of lung diseases that appear in small disease-specific areas. By combining CNNs and spatial attention mechanism, the CNN-Attention model can leverage the strengths of both CNNs and attention mechanism. The CNN part of the model can effectively extract local features and learn spatial representations from the input images. This enables the model to identify general patterns and abnormalities associated with lung diseases. The attention mechanism, applied with the CNN, enhances the model’s ability to focus on disease-specific regions within the images. Instead of treating the entire image equally, the spatial attention mechanism assigns higher weights to the regions that are most relevant for detecting the disease. Therefore, the model can learn to automatically attend to disease-specific areas within the input image, making it more effective in detecting and classifying lung diseases even in small localized regions.

Besides, applying the model on groups that shared the same clinical variables which is the view position leads to improve the AUC. Indeed, as presented in Table 3, for the classification of pneumonia, the Attention-based CNN model applied to all images without clustering (i.e. without differentiating between view positions) resulted in an AUC of 0.7941. However, after clustering the images based on their dominant view position, the model achieved a weighted AUC of 0.8176. Therefore, this statistical analysis revealed that the view position impacts the classification performance, improving the AUC by 2.35% for pneumonia classification.

Therefore, it is crucial to consider the view position when diagnosing lung diseases on the basis of radiographic images. In the medical context, the view position on radiographic images is fundamental for diagnosing lung diseases because it directly affects the visualization of lung structures and potential pathologies. PA views provide a clearer and more accurate representation of the lungs and chest cavity, as they minimize the magnification of the heart and other structures, allowing for a more precise evaluation of lung tissues. AP views, often used in bedridden patients or in emergency settings, can result in a distorted perspective, potentially obscuring or exaggerating certain lung pathologies. This can lead to challenges in detecting subtle abnormalities or accurately assessing the size and shape of lung lesions. Therefore, taking into account the view position when interpreting radiographic images is essential to ensure accurate assessment, avoid diagnostic errors and guarantee a consistent and standardized interpretation of results. This enables radiologists to optimize the detection of abnormalities, improve the quality of diagnoses, contribute to more effective treatment decisions and provide patients with more accurate and appropriate treatment.

The proposed approach was extended to the binary classification of consolidation and no finding (2751 images), as well as infiltration and no finding (20,049 images). Following the same methodology applied to pneumonia classification, two groups were obtained for each condition: one where the PA view was dominant and another where the AP view was dominant. To enhance image contrast, the Unsharp Masking method was applied with optimized parameters specific to each group and disease. Classification was then performed on both groups using the CNN and Attention-based CNN models. The best results were achieved using clustering, followed by Unsharp Masking and the Attention-based CNN model, with a weighted AUC of 0.8287 for consolidation and 0.7724 for infiltration. The classification results for each disease and the corresponding evaluation metrics detailed in Table 3, demonstrated the effectiveness of clustering followed by UM and the Attention-based CNN model. As clearly demonstrated in Table 3, applying the Attention-based CNN model directly to all images without clustering resulted in an AUC of 0.8035 for the classification of consolidation and and 0.7568 for the classification of infiltration. In contrast, performing classification after clustering on homogeneous groups sharing similar view position characteristic improved the AUC to 0.8287 for consolidation and 0.7724 for infiltration.

To further validate the results, additional evaluation metrics were computed for the configuration that yielded the best performance (clustering followed by UM, using CNN-Attention model). The results presented in Table 4 confirm the effectiveness of the proposed approach, achieving high accuracy in the binary classification of the three lung diseases: 0.7922 for pneumonia, 0.8063 for consolidation, and 0.7689 for infiltration. These findings highlight the importance of considering view position information when diagnosing lung diseases from radiographic images to improve classification performance. This enhanced ability to identify diseases is particularly valuable in the medical field, as it contributes to greater diagnostic precision and improved patient outcomes.

Visualizing attention allows researchers and physicians to better understand where the model is focusing its attention when classifying lung diseases. By overlaying the output of the attention layer with the original image, the most relevant areas contributing to the classification can be identified.

Figure 6 shows exemplary localization results of the proposed diagnosis model for pneumonia, consolidation and infiltration diseases. This visualization and localization enables the explainability of chest X-ray images and the interpretability of the model. It allows interpret the inner workings of the model and understand the specific parts of the image on which it focuses to perform classification. This can help build confidence in the model’s decisions, validate results and provide additional information for physicians when interpreting diagnostic results. To validate the results, these images were presented to a physician, who confirmed that the areas targeted by the model corresponded to the affected regions. This medical assessment reinforces the robustness of the proposed approach.

Additionally, the proposed approach was tested on additional lung diseases and compared with previous studies using the ChestX-ray14 dataset. According to Table 5, the results indicate that the proposed method achieves superior performance over the literature for the classification of all 14 diseases, with significant improvements. Therefore, considering the view position of CXR images, enhancing contrast and forcing the model to focus on the most informative regions through a spatial attention mechanism, leads to an improved ability to identify various lung diseases.

This study presents some limitations. First, using unsupervised clustering, some AP images were found as a minority within the PA cluster, and vice versa. These minority images may be atypical; their presence in a cluster with another view position suggests shared similarities. Grouping them with other images of the same view position could introduce variability. Therefore, implementing harmonization techniques to better align these images with others of the same view position type could potentially enhance classification performance. Another limitation relates to the attention mechanism. In this study, a spatial attention mechanism was employed to enable the model to focus on the most important regions of the image while ignoring irrelevant areas. Additionally, integrating channel attention could capture the relationships between channels in the input feature maps, emphasizing the most relevant feature channels. This dual attention mechanism could further enhance the performance of the classification model by addressing both channel and spatial dimensions.

## Conclusion

This paper evaluates the impact of clinical variables, specifically view position (AP/PA), on image attributes and interpretation, and consequently, on classification performance. The proposed approach begins by separating the images into clusters through unsupervised clustering. Clinical data within each cluster are then examined to determine whether specific clinical attributes predominate. The statistical analysis led to the identification of two groups: one group with a majority of PA views and another group with a majority of AP views. To enhance image contrast, the Unsharp Masking method was applied to each group. Furthermore, a spatial attention mechanism was integrated into the CNN model, allowing it to focus on the most relevant parts of the image for accurate lung disease classification. This proposed approach, developed separately for each view position, yields the highest weighted AUC for all the three lung diseases, exceeding literature by 4.06, 0.47 and 4.34% for pneumonia, consolidation, and infiltration classification, respectively. Additional evaluation revealed improved performance across all 14 diseases in the ChestX-ray14 dataset, highlighting the importance of considering the view position characteristic to improve classification performance.

Therefore, it is crucial to apply the model on groups with shared view position (AP/PA) to ensure the model is adapted to the specificity of each group. Indeed, considering the view position when diagnosing lung diseases on the basis of radiographic images leads to improve model’s performance, and therefore enables radiologists to improve the quality of diagnoses and contribute to more accurate treatment decisions. Besides, combining the strengths of CNNs and spatial attention improves the model’s performance in detecting disease-specific regions within the lungs by weighing features according to their importance for the classification task. Moreover, visualization provides a visual explanation of the lesion area and enables researchers and physicians to better understand where the model is focusing its attention when performing lung disease classification.

## Data Availability

The data that support the findings of this research are publicly available and are cited in the references.
